# Exploring the Genetic Landscape of Chorea in Infancy and Early Childhood: Implications for Diagnosis and Treatment

**DOI:** 10.3390/cimb46060337

**Published:** 2024-06-06

**Authors:** Giulia Spoto, Graziana Ceraolo, Ambra Butera, Gabriella Di Rosa, Antonio Gennaro Nicotera

**Affiliations:** 1 Unit of Child Neurology and Psychiatry, Department of Biomedical Sciences, Dental Sciences & Morpho-Functional Imaging, University of Messina, 98125 Messina, Italy; giulia.spoto27@gmail.com; 2 Unit of Child Neurology and Psychiatry, Department of Human Pathology of the Adult and Developmental Age “Gaetano Barresi”, University of Messina, 98125 Messina, Italy; graziana.c23@hotmail.it; 3 Unit of Child Neurology and Psychiatry, Department of Chemical, Biological, Farmaceutical & Environmental Science, University of Messina, 98125 Messina, Italy; butera.ambra@gmail.com; 4 Unit of Child Neurology and Psychiatry, Maternal-Infantile Department, University of Messina, 98125 Messina, Italy; antonionicotera@ymail.com

**Keywords:** chorea, choreiform, hyperkinetic movement disorder, movement disorder, Huntington’s disease, pediatric

## Abstract

Chorea is a hyperkinetic movement disorder frequently observed in the pediatric population, and, due to advancements in genetic techniques, an increasing number of genes have been associated with this disorder. In genetic conditions, chorea may be the primary feature of the disorder, or be part of a more complex phenotype characterized by epileptic encephalopathy or a multisystemic syndrome. Moreover, it can appear as a persistent disorder (chronic chorea) or have an episodic course (paroxysmal chorea). Managing chorea in childhood presents challenges due to its varied clinical presentation, often involving a spectrum of hyperkinetic movement disorders alongside neuropsychiatric and multisystemic manifestations. Furthermore, during infancy and early childhood, transient motor phenomena resembling chorea occurring due to the rapid nervous system development during this period can complicate the diagnosis. This review aims to provide an overview of the main genetic causes of pediatric chorea that may manifest during infancy and early childhood, focusing on peculiarities that can aid in differential diagnosis among different phenotypes and discussing possible treatment options.

## 1. Introduction

Chorea is a hyperkinetic disorder characterized by excessive, irregular, involuntary movements that appear variable in speed, duration, and direction. The involvement may be focal or generalized, and a sequence of choreic movements may flow from one muscle group to another, affecting the limbs, trunk, neck, face, and tongue. Ballism is a particular type of chorea involving the proximal joints and consisting of rapid movements of large amplitude with a flinging quality [1].

Chorea impairs normal motor function and may be worsened by movement, attempts at movements, or stress; however, these movements can become part of intentional actions (namely “parakinesias”) but cannot be voluntarily suppressed, and usually persist even during relaxation, resulting in constant motion [1,2]. The presentation may be paroxysmal if the movement disorder is characterized by recurrent attacks of abnormal movements or chronic if it shows a static or progressive course [3].

Addressing chorea is a challenging task, since multiple hyperkinetic movement disorders can co-occur in the same patient, with considerable overlap [4]. Furthermore, it can be particularly difficult in very young children. For example, transient motor phenomena resembling chorea may occur during infancy, given that it is a period of intense nervous system development [5]. For instance, between 9 and 20 weeks, infants show a pattern of spontaneous, continuous, involuntary, multidirectional, and small movements of moderate speed and variable acceleration involving the neck, trunk and limbs, called fidgety movements [6]. These movements can become more forceful, abrupt, faster, and sometimes somewhat ballistic, in the setting of excitement [5]. Peculiarly, in patients with severe vision impairment and motor developmental delay, they can be exaggerated in amplitude and jerky in character, lasting until 8 to 10 months post-term age [7]. In addition, children aged between 6 and 12 months may present rapid, unpredictable movements resembling chorea, occurring mainly in the upper limbs and mostly during anger or frustration [4].

Although the neurophysiological mechanisms behind chorea are not entirely clear, it is commonly accepted that this movement disorder emerges from alterations affecting the basal ganglia and their connections, including the ones connected to the cerebral cortex, cerebellum, and thalamus [1,2]. Focal lesions of the striatum and/or dysregulation of medium spiny neurons, accounting for approximately 95% of striatal cells and forming its output projections, have been highly implicated in the etiopathogenesis of chorea [8,9]. Indeed, structural lesions on one side of the basal ganglia can give rise to hyperkinetic movements restricted to the contralateral side of the body, defined as hemichorea or hemiballism [10]. Several acquired causes have been linked to pediatric chorea, and most cases are considered secondary to known or presumed injury [2,9]. Nevertheless, thanks to the recent advances in DNA sequencing techniques, the list of genetic etiologies underlying choreic movements is rapidly increasing. Among them, pediatric-onset chorea may be frequently associated with other neurological disorders (i.e., spasticity, ataxia, intellectual disability (ID), and epilepsy/epileptic encephalopathy) or be part of a complex phenotype that involves systemic signs and symptoms [4,11,12].

Herein, we aim to provide an overview of the main genetic causes of pediatric chorea that may manifest during infancy and early childhood (within 5 years of age), focusing on peculiarities that can aid in differential diagnosis among different phenotypes. In this context, we have categorized the genes based on different presentations, considering whether chorea is the primary feature of the disorder (primary chorea), if it is part of epileptic–dyskinetic encephalopathy, or associated with multisystemic involvement. Additionally, we have taken into account whether the hyperkinetic disorder is a persistent condition (chronic chorea) or paroxysmal (paroxysmal chorea). Finally, we have explored the literature and discussed the possible treatment options available.

## 2. Primary Chorea

**Figure 1:**
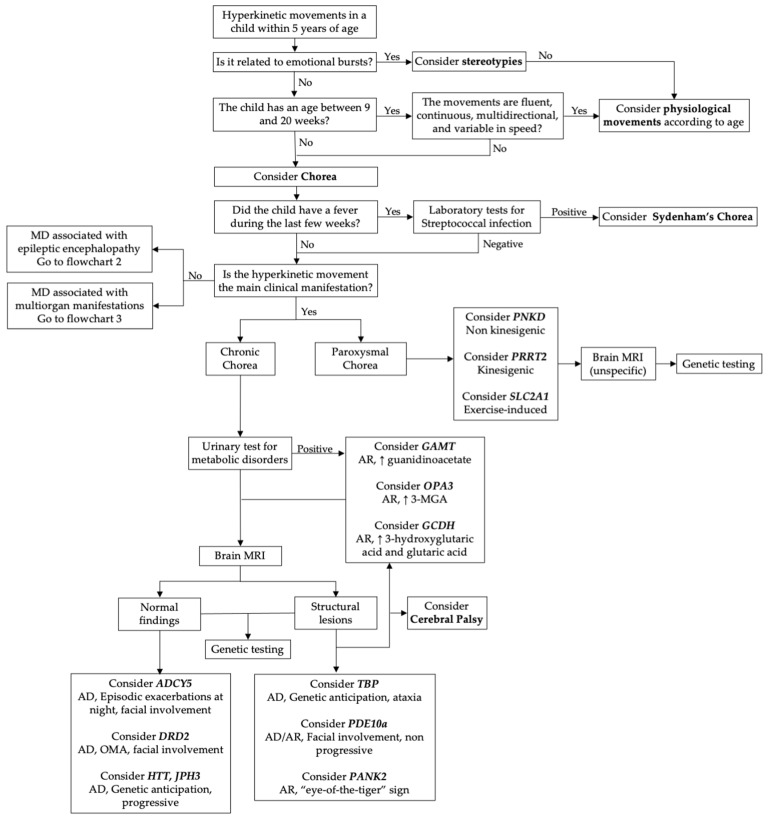
Diagnostic flowchart of primary chorea. Legend: 3-MGA = 3-methylglutaconic acid; AD = autosomal dominant; AR = autosomal recessive; MD = movement disorder.

In this section, we have explored the genes involved in the onset of chorea or choreic movements as primary symptoms of the disease. Figure 1 shows a diagnostic flowchart to orient the clinicians in the genetic research.

### 2.1. Chronic Chorea

Chronic chorea, as the predominant symptom of the disease condition, is mainly related to pathogenic variants in genes responsible for post-receptor intracellular signaling (i.e., *PDE10A* and *ADCY5*) and encoding for proteins with enzymatic or receptor function, such as *OPA3*, *GAMT*, *GCDH*, and *DRD2*. *PDE10A* and *ADCY5* genes play a crucial role in the physiology of movement control by modulating striatal cyclic nucleotide levels [13]. Cyclic adenosine monophosphate (cAMP) activity is determined by the balance between synthesis and degradation, controlled by adenylyl cyclase 5 (ADCY5) and phosphodiesterase 10A (PDE10A), respectively. cAMP plays a prominent role as a second messenger in the striatal medium spiny neurons (MSNs), finely regulating movement execution; moreover, alterations in its turnover may result in hyperkinetic movement disorders [14].

*ADCY5* is highly expressed in the basal ganglia, in particular the nucleus accumbens, olfactory tubercle, and MSNs, where it is involved in motor function and cortical arousal by regulating dopaminergic signaling through the two dopamine receptors—stimulating D1 and inhibiting D2 [15]. *ADCY5*-related disease is genetically heterogeneous, with most cases presenting as gain-of-function (GOF) mutants [16]. However, loss-of-function (LOF) mutants may also be the pathophysiological mechanism involved [17]. *ADCY5*-related dyskinesia is an autosomal dominant disorder characterized by infantile-to-childhood-onset mixed hyperkinetic movement disorder [15]. The clinical phenotype includes variable combinations of hyperkinetic movements, most commonly focal or generalized chorea, dystonia, and myoclonus. Choreic movements also classically involve the facial muscles (perioral and periorbital), with episodic exacerbations, variability in severity, and a duration from minutes to hours, observed more frequently upon night-time awakening, with subsequent delayed sleep [18].

Dystonia (which can be painful or limit mobility), myoclonus, and paroxysmal hyperkinetic movements (sometimes referred to as non-epileptic ‘ballistic bouts’) triggered by emotions/anxiety/stress are different presentations of the disorder; moreover, dyskinesia can affect the tongue, resulting in speech impairment [19,20,21].

Patients with *ADCY5*-related disorder may have developmental delay, axial hypotonia, and normal to mild ID. Although *ADCY5*-related disorder is a non-progressive condition, the chorea and ballistic episodes are thought to improve during adolescence, with the phenotype changing to dystonia and myoclonus in adulthood. Neuroimaging is usually normal [9].

*PDE10A* encodes phosphodiesterase 10A, which regulates cGMP and cAMP degradation in MSNs [13]. Dominant and recessive *PDE10A* variants have been shown to lead to a loss of enzymatic function or reduced striatal protein levels [22]. Patients with heterozygous variants of *PDE10A* presented with early-onset generalized chorea with the occasional involvement of drooling, dysarthria, and orolingual dyskinesia [23]. Biallelic *PDE10A* variants are associated with a more severe phenotype, presenting at an earlier age of onset of chorea (within 6 months), with markedly delayed motor and language milestones, axial hypotonia, and severe dysarthria; furthermore, in some cases, mild ID was also reported. Moreover, a single patient showed childhood-onset epilepsy [13]. Despite the more severe neurological involvement, brain magnetic resonance imaging (MRI) is usually normal [13]. In some cases, characteristic symmetrical T2-hyperintense bilateral striatal lesions have been reported [22,23]. The disease course seems to be non-progressive, although diurnal fluctuations are more severe upon awakening in the morning [9,24].

The GOF variant in *DRD2*, the gene encoding the dopamine receptor 2 (D2R), has been linked to a mixed phenotype of chorea and dystonia. The D2R is well established in the regulation of movement. For example, the overstimulation of G-protein-dependent signaling by the mutant D2R is associated with reduced arresting recruitment, and activation may be the cause of the hyperkinetic movement disorder [25]. The subjects with the mutant D2R showed early-onset choreatic movements of the orofacial muscles and generalized to trunk and all extremities, occasional myoclonus involving the neck and upper limbs, dystonic posturing of the head, and oculomotor apraxia [25,26]. The course of disease is progressive, associated with global developmental delay and axial hypotonia. The brain MRI is usually normal [26]. *DRD2* has also been linked to psychiatric symptoms such as generalized anxiety, panic attacks, and attention deficit hyperactivity disorder [25,26].

Pathogenic variants of *OPA3* have been reported in Costeff syndrome (CS). It is a rare neurogenetic disorder, with an autosomal recessive inheritance, characterized by the development of optic atrophy, ataxia, increased urinary excretion of 3-methylglutaconic acid (3-MGA), choreoathetosis, dystonia, and spastic paraparesis in early childhood [27,28]. This condition is predominantly observed in individuals of Iraqi-Jewish descent and, to date, approximately 50 patients have been described [29]. The first signs of neurological deficit appear in infancy or early childhood, with developmental delay, choreiform movements, ataxia, and visual disturbances) [28]. The dominant motor presentation may vary in severity but does not seem to worsen with age. The chorea usually involves the face, neck, and limbs [28,30]. Usually, the intelligence quotient is within the normal range, but a mild cognitive impairment may occur in the second decade of life [31]. Although cerebellar or optic chiasm atrophy have been described in a few patients, usually, brain MRI findings are not informative. Seizures are an atypical feature of CS [31].

Among the other genes associated with chronic chorea, the *GAMT* gene is responsible for an autosomal recessive disorder characterized by cerebral creatine deficiency and an accumulation of guanidinoacetate in tissues and body fluids. The clinical features include ID, severe speech delay, autistic-like behaviors, and epilepsy. A movement disorder has been reported in 50% of patients before 12 years of age, with one case showing the onset at 4 months [32,33].

Mixed movement disorder, including chronic chorea, may also be present in glutaric aciduria type 1 (GA1), an autosomal recessive condition caused by pathogenic variants of the glutaryl CoA dehydrogenase (*GCDH*) gene. The estimated incidence of GA1 is 1/30,000–110,000 worldwide [34]. This neurometabolic condition is characterized by impaired catabolism of lysine, hydroxylysine, and tryptophane, with a consequent accumulation of 3-hydroxyglutaric acid and glutaric acid, leading to neuronal death through excitotoxicity [35]. It has a poor prognosis, due to metabolic crises and progressive deterioration. In countries without newborn screening, clinical suspicion is essential for diagnosing cerebral organic aciduria [34]. In most patients, the mixed movement disorder presents with generalized dystonia and occasional generalized chorea. Prominent orofacial involvement is a consistent feature in patients with movement disorders, resulting in speech impairment characterized by combined hyperkinetic dysarthria and speech apraxia [35].

Chorea is also a prominent feature of the pantothenate-kinase-associated neurodegeneration (PKAN), a subtype of neurodegeneration with brain iron accumulation (NBIA), a group of diseases characterized by iron accumulation in the basal ganglia [36]. PKAN is associated in most cases with variants of the pantothenate kinase 2 (*PANK2*) gene, which encodes for a protein that belongs to the pantothenate kinase family and is the only pantothenate kinase located in the mitochondria [37,38]. This condition affects approximately one to three people per 1,000,000 [39]. Two courses of PKAN have been distinguished: the classic early-onset and the atypical late-onset variety. Particularly, the symptoms of the classic form include dystonia, speech difficulties, spasticity, and choreoathetosis that emerge within 6 years of age (usually around 3 years). Gait and postural difficulties are common features at the onset of the disease, and neurological examination may detect the involvement of the corticospinal tract, with spasticity, hyperreflexia, and extensor toe signs. The progression of the disorder is rapid, and death usually occurs at a young age [40]. The brain MRI is characteristic and shows the “eye-of-the-tiger” sign, a T2-hypointensity of the globus pallidus with a central hyperintensity, corresponding to excessive brain iron accumulation [41].

Among the genetic causes of chorea, some inherited forms are related to the expansion of trinucleotide repeats that may be characterized by the so-called “genetic anticipation”, in which longer repeats are associated with an earlier onset of symptoms. Huntington’s disease (HD) is the most common cause of chronic progressive chorea in adults. HD is an autosomal dominant neurodegenerative disorder caused by a pathological CAG repeat expansion in the gene *HTT*, located in chromosome 4p, and coding for huntingtin [10,42]. Huntington’s disease stands as the most prevalent genetically determined neurodegenerative condition, affecting at least 12.4 out of every 100,000 individuals [10]. In the journey of this disease, the duration after motor onset spans approximately 18 years for adolescent and adult patients, whereas, for those with juvenile onset, this duration shortens to a span of 9 years. The mean age of onset is 45 years old, but 4% to 10% of patients present with juvenile-onset HD (JHD) before the age of 20, with possible childhood onset < 10 years. The length of the CAG expansion is the most relevant determinant of the age at the onset and progression of disease, particularly when the repeat expansion is inherited through the father [9]. Genetic anticipation also occurs in other repeat disorders, including some of the HD-like disorders. Although chorea is the initial and predominant motor sign in 80% of adult-onset HD, it is rarely seen in early JHD, which is characterized in the early stages by bradykinesia, rigidity, dystonia, and parkinsonian features. Chorea, if present, is less prominent, but gradually develops as the disease progresses in a subset of adolescent HD [42,43]. In the early stages, patients may also present with cognitive dysfunction and personality changes or psychiatric symptoms.

About 1% of suspected HD cases emerge as phenocopy syndromes, the so-called HD-like (HDL) syndromes. Among these syndromes, a childhood onset of chorea has been reported in HDL2, HDL3, and HDL4. HDL2 is caused by a CAG repeat expansion of the junctophilin-3 (*JPH3*) gene on chromosome 16q24.2. HDL2 is a rare condition, with fewer than one hundred cases worldwide, almost exclusively affecting individuals of African ancestry. It has been primarily documented in South Africa, the Americas, the Caribbean, and Europe, including cases from Brazil and Venezuela. All reported cases have confirmed or likely African ancestry [10,44]. Due to genetic anticipation, the age of onset is inversely related to the size of the CAG repeat expansion, especially in the case of maternal inheritance [42]. HDL3 is an autosomal recessive neurodegenerative disease, described only in one Saudi Arabian family and caused by a mutation mapped to chromosome 4p15.3. The clinical phenotype with early-childhood onset is characterized by chorea, dystonia, ataxia, intellectual impairment, and developmental delay [2,42]. HDL4, also classified as spinocerebellar ataxia type 17 (SCA17), is an autosomal dominant disorder caused by trinucleotide repeats expansion in the TATA-box-binding protein (*TBP*) gene located on chromosome 6q27. Similar to HD, there is a negative correlation between the size of repeat expansion and the age of onset. The clinical phenotype is characterized by predominant cerebellar ataxia, accompanied by another movement disorder that may be progressive or paroxysmal, such as chorea, dystonia, tremor, or parkinsonism [44]. While it is frequently observed in Japan, Germany, and Italy, its minimal prevalence in northeastern England was estimated at 0.16/100,000 [45]. The life expectancy is not clearly defined in the literature, but some research groups have reported cases of premature death before the age of 50 [46,47]. Neuropsychiatric symptoms (psychosis and dementia) and migraine are often the initial manifestations, but a variety of pyramidal and extrapyramidal motor manifestations have been described [2]. Table 1 summarizes the clinical features, brain imaging, and treatment options of the genetic disorders in which chronic chorea is the prominent symptom of the condition.

### 2.2. Paroxysmal Chorea

Paroxysmal chorea is characterized by brief sudden episodes of rapid and disordered involuntary movements, known as choreiform movements. However, in most cases, these choreiform movements are associated with other transient episodes of abnormal movements, including dystonia, ballism, myoclonus, ataxia, or a combination of them. Due to the frequent co-occurrence and challenging delineation of these hyperkinetic disorders, some authors have proposed categorizing them under the umbrella term “Episodic or Paroxysmal Hyperkinetic Movement Disorders”, dividing them into two groups: paroxysmal dyskinesia and episodic ataxia [48,49]. Paroxysmal dyskinesia, characterized by the occurrence of transient hyperkinetic movements, can be subdivided into several syndromes based on triggering factors, as follows: paroxysmal kinesigenic dyskinesia (PKD) or choreoathetosis, paroxysmal non-kinesigenic dyskinesia (PNKD), paroxysmal exercise-induced dyskinesia (PED), and paroxysmal hypnogenic dyskinesia (PHD). In these conditions the neuroimaging is usually uninformative and the diagnosis rest on clinical features and genetic testing (see flowchart in Figure 1) [48].

PKD is an inherited or acquired disorder characterized by attacks of chorea and/or dystonia with no loss of consciousness or pain, lasting less than 1 min. Attacks are triggered by movements or acceleration of ongoing movements, but may also be precipitated by sleep deprivation and emotions, such as anxiety/startle. Some patients report a sensory aura or premonitory sensation before the attack [50,51]. PKD may also be a feature of more complex chronic neurological disorders [48,49]. The gene thought to be responsible for PKD has been identified as the proline-rich transmembrane protein 2 (*PRRT2*), which may also be involved in other paroxysmal disorders [52,53]. It is an autosomal dominant condition, in which male patients far outnumber female patients. A family history of a form of epilepsy called benign familial infantile seizure or paroxysmal movement disorders is frequent in these patients [48,54]. Typically, attacks can affect the face, trunk, and limbs, starting on one side, with a tendency of generalization. The frequency of attacks ranges from hundreds per day to one or two per year, but it tends to decrease with age and may even disappear completely in adulthood, regardless of any treatment [49,51]. Several other genes have been associated with PKD, and include *PNKD*, *SCN8A*, *SLC2A1*, and *SLC16A2* pathogenic variants [48].

PNKD is a rare autosomal dominant disorder with infancy or early childhood onset, caused by variants of *PNKD*, previously known as the myofibrillator regulator-1 (*MR-1*) gene. PNKD episodes, consisting of chorea, dystonia, or both, are less frequent than PKD, ranging from 1 min to 12 h, and are triggered by caffeine, alcohol, stress, and fatigue. Attacks may occur spontaneously at rest and may be focal with or without generalization [48,49]. Sometimes, patients have reported premonitory feelings, including weakness, shortness of breath, and migraine [49]. The frequency may regress with age. There is no associated epilepsy or neurodevelopmental disorder [55].

PED attacks consisting of dystonic and/or choreic episodes are triggered by sustained exercise, but also by stress, sleep deprivation, or, more rarely, by cold, muscle vibration, or passive movements. These usually last 5–30 min (rarely up to 2 h) [48,51]. *SLC2A1* variants are the most common causative defect but are also associated with PKD and PNKD phenotypes [48]. The *SLC2A1* gene encodes the glucose transporter type 1 (GLUT1), and pathogenic variants lead to a broad spectrum of neurological disease, including developmental and epileptic encephalopathy. The large majority of patients have focal/unilateral involvement, and generalization is rather unusual. During the attacks, some patients experienced oculogyric crises, gait disturbances, clumsiness, weakness, and migraines [48,49]. The attacks ranged from several per day to one per month and lasted between 15 and 40 min [48,51].

PHD is the least common phenotype of paroxysmal disorders. It is characterized by attacks occurring during non-rapid eye movement sleep (NREM) sleep and includes dystonic posturing and ballistic or choreic movements, without EEG abnormalities [56]. PHD is a core feature of *ADCY5* and *PRRT2* variants, and the attacks last for less than 1 min in most cases [51].

The *ADCY5*, *PDE10A*, *PDE2A*, and *GNAO1* genes have been related to different forms of paroxysmal dyskinesia without a prevalent phenotype [48].

Similarly, the pathogenic variants of the genes involved in channelopathies have been described in patients with PKD or PNKD phenotypes. Particularly, GOF variants of *KCNMA1* have been reported to cause both early-onset PNKD and PKD, associated with epilepsy or developmental delay [48]. *KCNA1* heterozygous variants have also been found in epileptic encephalopathy, epilepsy, myokymia, and PKD, while homozygous variants have recently been shown to cause epileptic dyskinetic encephalopathy [48,56]. GOF mutations of *CACNA1A* are associated with developmental and epileptic encephalopathy, epilepsy, and familial hemiplegic migraine, while LOF variants may occur in paroxysmal movement disorders, including PKD and PED [48,51].

## 3. Chorea and Epileptic–Dyskinetic Encephalopathy

**Figure 2:**
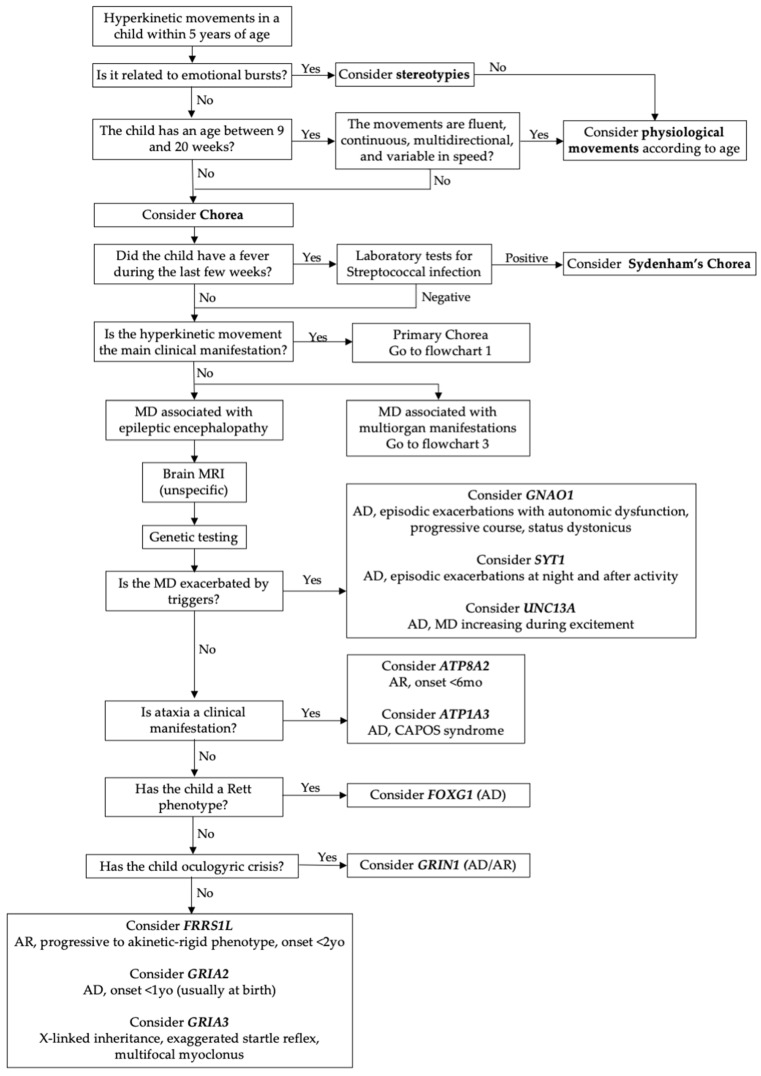
Diagnostic flowchart of epileptic–dyskinetic encephalopathy. Legend: AD = autosomal dominant; AR = autosomal recessive; CAPOS = cerebellar ataxia, areflexia, pes cavus, optic atrophy, and sensorineural hearing loss; MD = movement disorder; yo = years old.

Epileptic–dyskinetic encephalopathies are an emerging group of clinically heterogenous disorders characterized by early-onset developmental and epileptic encephalopathy associated with hyperkinetic movement disorders [9,57]. The genes related to these conditions were initially reported in severe epileptic encephalopathies; however, the increasing application of genetic techniques has allowed us to broaden the genotype–phenotype correlation, revealing that they can also be related to isolated movement disorders (most frequently chorea) [9]. Figure 2 shows a diagnostic flowchart regarding these disorders. Most of these genes are involved in the early phases of brain development or synaptic transmission activity [58].

The movement disorder is considered a prominent feature in some of these conditions, such as the Forkhead Box G1 (*FOXG1*)-related disorders [59]. This gene encodes for a transcriptional repressor that plays a crucial role in telencephalon development during fetal period [60]. It is also involved in regulating proliferation, neurogenesis, differentiation, and neurite outgrowth in the cerebral cortex, basal ganglia, and hippocampus [4,61,62]. Patients with severe truncating variants usually present with the onset during infancy, establishing a clinical phenotype called “congenital variant of Rett syndrome”. This condition is characterized by severe developmental encephalopathy, acquired microcephaly, profound ID with no language skills, epilepsy, and a hyperkinetic movement disorder [63]. However, individuals with missense variants may show a milder phenotype, with independent ambulation, spoken language, normal orbito-frontal circumference, and the ability to use their hands [64]. The movement disorder can emerge within the first year of life and encompasses different combinations of chorea (mainly involving the upper limbs), orolingual/facial dyskinesias, dystonia, myoclonus, and hand stereotypies [57,60]. Particularly, chorea represents the most frequent movement disorder in *FOXG1*-related conditions, followed by orolingual/facial dyskinesias [64]. Neuroimaging can display corpus callosum hypoplasia or aplasia, delayed myelination, simplified gyration, and frontotemporal abnormalities [57].

Similarly, variants of the *GNAO1* gene have been associated with a heterogeneous phenotype spectrum ranging from a catastrophic developmental and epileptic encephalopathy to a progressive choreic movement disorder in the absence of epilepsy [65,66]. *GNAO1* encephalopathy is mainly characterized by movement disorders (incidence > 80%). The main causes of death include respiratory complications and dystonic status, with fever and infections as the main triggering factors [67]. The gene encodes the α-subunit of heterotrimeric guanine-nucleotide-binding protein G (Gαo), which is regarded as the most abundant membrane protein in the mammalian central nervous system and represents about 1% of total brain membrane protein [68]. Gαo acts as a modulator of cAMP and is involved in cytoskeletal remodeling and the functional polarity of developing neurons, thus, in the regulation of synaptic function and neuronal excitability [69,70]. Although a genotype–phenotype correlation has not been established yet, recent research has suggested that Gαo function can be altered by different mechanisms related to the variants [66]. LOF variants have been associated with a severe form of early infantile epileptic encephalopathy. At the same time, GOF mutations have been related to hyperkinetic disorders (i.e., chorea, ballismus, dystonia, and orofaciolingual dyskinesia) with or without epilepsy [57,71]. Particularly, it has been proposed that the modulation of calcium channels and synaptic vesicle release may be critically involved in the pathophysiology of the movement disorder [71].

The movement disorder is present in almost 80% of individuals affected by the *GNAO1*-related disorder, and symptoms such as developmental delay, hypotonia, prominent chorea, and dystonia have been described in approximately 50% of cases with a typical age at onset of four [60,71]. Although, initially, the motor phenotype can be nonspecific, the clinical course is severe and fluctuating, characterized by episodic exacerbations of chorea and ballism combined with autonomic dysfunction. These episodes can be spontaneous or triggered by infections and other stressors (i.e., high temperature and emotions) and may persist for hours or days, representing a life-threatening condition and requiring admission to intensive care units for sedation [4,57,66,71]. It is noteworthy that the occurrence of this emergency has been associated with the choreo-athetosic phenotype [71]. Interestingly, in some patients with a severe phenotype, the mothers described increased fetal movements, which have been interpreted as dyskinetic episodes [66]. Brain imaging is often inconclusive for the diagnosis, as normal or unspecific brain MRIs have been reported [66,71].

The other genes involved in the onset of epileptic–dyskinetic encephalopathies include the ones encoding for subunits of the ionotropic glutamate receptors [57,72,73,74]. *GRIN*-related disorders are typically referable to LOF or GOF de novo mutations in *GRIN1*, *GRIN2A*, *GRIN2B*, *GRIN2C,* or *GRIN2D*, and the phenotypes may vary considerably depending on the effect of the underlying pathogenic variants on N-methyl-D-aspartate (NMDA) receptor function [75]. Hyperkinetic movements have been described as prominent features in *GRIN1*-related disorders, with a choreic, dystonic, or dyskinetic phenotype, including characteristic oculomotor abnormalities such as oculogyric crises [72,76]. The other common symptoms of this disorder are severe ID and epilepsy [2]. *GRIN1* encodes for the GluN1 subunit, representing a ubiquitous receptor component. Most *GRIN1*-related movement disorders are caused by de novo LOF mutations that affect the trans-membrane domains of GluN1, which is a highly conserved region [72]. Similarly, though they are not the prominent feature, choreic movements have been described in patients with developmental and epileptic encephalopathy caused pathogenic variants of *GRIN2D*, which encodes for the GluN2 subunit of the NMDA receptor [77].

The pathogenic variants in glutamate ionotropic alpha-amino-3-hydroxy-5-methyl-4-isoxazolepropionic acid (AMPA) receptors, such as *GRIA2* and *GRIA3* genes, have also been associated with movement disorders. Particularly, chorea or choreoathetoid movements with an onset within the first year of age have been described in patients with *GRIA2* pathogenic variants [73,78]. *GRIA2*-related encephalopathy is characterized by early-onset developmental and epileptic encephalopathy, complex movement disorders (including chorea, dystonia, and dyskinesia) with or without epilepsy, varying combinations of tone abnormalities at birth, and neurobehavioral and/or psychiatric disorders [73]. Moreover, chorea has been reported with an X-linked inheritance in patients with *GRIA3* pathogenic variants. The phenotype includes mild ID, dysarthria, and a tightly consistent movement disorder starting from birth onwards and combining an exaggerated startle reflex with multifocal myoclonus and generalized chorea [74].

AMPA glutamate receptor signaling is also implicated in LOF mutations of the ferric chelate reductase 1-like (*FRRS1L*) gene, which encodes an accessory protein that is an outer component of the receptor and facilitates its action [2,4]. Its main phenotype includes developmental regression, severe ID, epileptic encephalopathy, and severe chorea [79]. In these patients, a normal early development is described, with a regression that occurs within 2 years of age, followed by the onset of epilepsy, psychomotor regression, and progressive movement disorder characterized by choreoathetosis and ballismus [2,4]. The impairment of glutamatergic neurotransmission caused by LOF pathogenic variants might underlie the epileptic–dyskinetic encephalopathy [79].

Chorea can also be caused by de novo pathogenic variants in genes essential for the release of neurotransmitters through the fusion of synaptic vesicles, such as *SYT1* and *UNC13A*. *SYT1* encodes synaptotagmin-1, the primary cerebral isoform, that modulates synaptic vesicle fusion by binding calcium and has key role in developmental of motor control and cognitive abilities [80]. Subjects with pathogenetic variants of *SYT1* present with childhood-onset hyperkinetic generalized movements, including dystonia and predominant chorea (especially of the lower limbs), accompanied by severely delayed motor development and profound ID [81]. Similar to patients with *ADCY5*-related dyskinesias, episodic exacerbations of abnormal movement are maximal at night and worsen before and after purposeful motor activity [81]. Symptom severity ranges from dystonic posturing and mild chorea to severe mixed movement disorder with vocal dystonia and ballism [80].

The presynaptic protein munc13-1 (*UNC13A* gene) is essential for vesicular priming and the generation of the readily releasable pool of vesicles. A de novo variant of *UNC13A* produced a clinical phenotype similar to that described for *SYT1* [82]. Munc13-1 variants cause a GOF, leading to increased synaptic release and abnormal plasticity, resulting in hyperkinesia. One patient showed an early-onset and continuous form of dyskinesia with choreoathetosis, intention tremor of the arms increasing during excitement, and limited fine motor skills. Moderate global developmental delay and neuropsychiatric changes, with mild ID, autism spectrum disorder, and comorbid attention deficit disorder were also present [82].

Choreic movements with an onset within infancy and early childhood have also been described in patients with pathogenic variants of genes encoding for cellular transporters [83,84,85]. The *ATP1A3* gene encodes the α3 isoform of the catalytic subunit of the Na^+^/K^+^ pump responsible for maintaining electrochemical gradients for Na^+^ and K^+^ across the plasma membrane. Notably, the α3 subunit is selectively expressed in neurons [4]. Dominant *ATP1A3* pathogenic variants have been related to three specific neurological phenotypes: dystonia, alternating hemiplegia of childhood, and CAPOS (cerebellar ataxia, areflexia, pes cavus, optic atrophy, and sensorineural hearing loss) syndrome [83]. In a patient of 17 months of age, a de novo pathogenic variant in *ATP1A3* (c.2266C>T; p.Arg756Cys) has been related to a phenotype characterized by an episode of generalized hypotonia and flaccid paralysis that lasted for a few weeks, followed by persistent choreoathetoid movements [86]. Similarly, a patient with a de novo heterozygous variant of *ATP1A3* (c.2452G>A; p.Glu818Lys) causative of CAPOS syndrome showed a fever-induced episode of marked chorea, ataxia, flaccid tetraplegia with areflexia, and nystagmus at the age of four years [84].

*ATP8A2* encodes a protein that actively transports phospholipids across cell membranes. Eleven individuals with biallelic variants of *ATP8A2* have been described, all consistently showing global developmental delays, severe hypotonia, optic atrophy, and chorea, with symptoms onset within 6 months of age. Feeding difficulties have been reported in most of these patients, and other hyperkinetic movement disorders, such as dystonia and orofacial dyskinesia, may be additional features [85].

Other genes associated with epileptic–dyskinetic encephalopathies include *CDKL5*, *SCN1A*, *SCN2A*, *ARX*, *GABRA2*, *ALG13*, and *RHOBTB2*. However, chorea or choreic movements are not considered the predominant symptom and usually occur later, while an onset before 5 years of age has only been described in single patients. For this reason, their description lies beyond the scope of this review. Clinical features, brain imaging, and treatment options of the epileptic–dyskinetic encephalopathies associated with chorea are summarized in Table 2.

## 4. Chorea Associated with Other Multiorgan Manifestations

**Figure 3:**
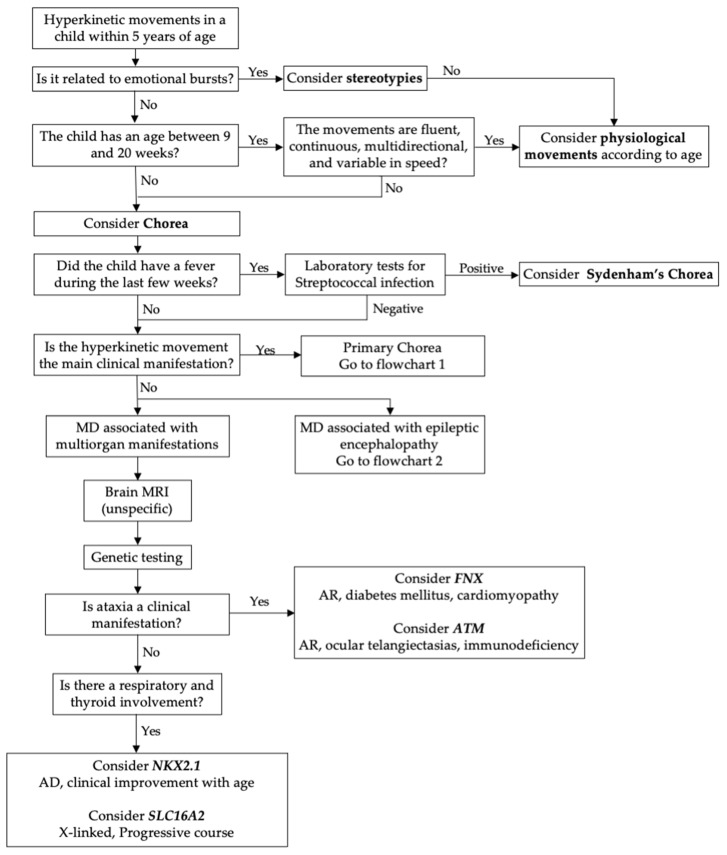
Diagnostic flowchart of the chorea with multiorgan manifestations. Legend: AD = autosomal dominant; AR = autosomal recessive; MD = movement disorder.

The literature identified conditions linked to variants of the *NKX2-1*, *SLC16A2*, and *ATM* genes as causes of the chorea phenotype associated with multisystem impairment. Figure 3 shows a diagnostic flowchart of the choreic disorders, including multiorgan manifestations.

*NKX2-1*-related disorder, or benign hereditary chorea (BHC), is a rare disorder encompassing heterogeneous clinical features, characterized by childhood-onset chorea, combined with other neurological, pulmonary, and endocrinological abnormalities [87]. It is caused by defects in the NK2 homeobox 1 (*NKX2.1*) gene, previously named thyroid-specific transcription factor-1 (*TITF1*), located in chromosome 14q13. *NKX2.1* is involved in the embryogenesis of brain, lung, and thyroid tissue. *NKX2.1* variants have been described in patients with variable combinations of congenital hypothyroidism, pulmonary dysfunction, and chorea [88]. Chorea in BHC may start in infancy or early childhood (<1 year) and is often accompanied by development delay and hypotonia. Chorea is usually generalized, with a non-progressive course, and may improve in adulthood. The movement disorder may be worsened by stress and excitement, improved by alcohol, and relieved by sleep [60].

A parallel disorder described as a cause of BHC and thyroid dysfunction is Allan–Herndon–Dudley syndrome (AHDS), an X-linked disorder caused by pathogenic variants of the *SLC16A2* gene. It is considered a rare disease, and approximately 200 individuals have been reported in the literature [89]. Like BHC, AHDS is associated with chorea and dystonia, but, in this case, the movement disorder may be present at birth or develop within the first 6 months of life. The movement disorder can develop into spastic paraplegia during the first two decades of life [2].

Ataxia-telangiectasia (A-T) is a multisystem disease characterized by progressive neurological dysfunction, immunodeficiency, and a predisposition to malignancy. It is caused by *ATM* gene variants, encoding a serine–threonine kinase, which plays a critical role in DNA repair [44]. The prevalence of patients with A-T in Europe is estimated to be 1 in 150,000. The majority of individuals with A-T experience onset of the disease during early childhood and, unfortunately, often face the prospect of malignancies or respiratory failure by their second or third decade of life. The morbidity and mortality observed in A-T patients are closely tied to their specific *ATM* genotype [90]. Neurological impairment may present in 1–4 years with progressive ataxia, oculomotor apraxia, telangiectasias, and involuntary movements such as choreoathetosis, or a variable combination of dystonia, chorea, and myoclonus. Chorea is more common than dystonia in childhood and may precede ataxia [57].

Friedreich ataxia is the most common autosomal recessive ataxia in Caucasian populations, caused by homozygous GAA triplet repeat expansions in exon 1 of the frataxin gene (*FXN*) [44]. Friedreich’s ataxia has a prevalence of approximately 1 in 50,000 in Caucasian populations and is particularly frequent in Cyprus and the Rimouski area of Quebec. Population studies have highlighted clinical differences between ethnicities and geographical regions. French-Canadian patients show clinical differences compared to the Acadians of Louisiana, with the latter exhibiting slower peripheral involvement and a lower incidence of cardiomyopathy, resulting in a longer life expectancy [91]. An animal model study suggested that the ubiquitous reduction in frataxin in Drosophila leads to a reduced lifespan, increased sensitivity to oxidative insults, neurodegenerative effects, and severe impairments in locomotor activity [92]. The clinical syndrome is characterized by progressive ataxia, dysarthria, limb weakness, impaired proprioception and vibration sense, areflexia, and extensor plantar responses. Patients with heterozygous repeat expansion may have atypical clinical features, such as generalized and progressive chorea, with early-childhood onset. However, chorea has also been reported in patients who are homozygous for the expansion [93]. Cardiomyopathy, diabetes, scoliosis, and hearing and vision impairment are also part of the clinical phenotype [94]. Table 3 shows a synthetic overview of clinical features and treatment options of conditions presenting with chorea and systemic manifestations.

## 5. Treatment Options

The treatment options for pediatric chorea encompass both symptomatic management and therapies targeting the underlying causes. To date, there are no universally accepted guidelines and, though etiology-specific treatment is the desirable choice, it is currently available only for a minority of conditions [95]. A ketogenic diet supplemented with L-carnitine is considered the standard treatment of Glut1 deficiency, but permissive KD, in which fat represents only 60–70% of the daily caloric intake, have also been proposed. These include the modified Atkins diet (the most used), the medium-chain triglyceride diet, and low glycemic index treatment, which have been proven effective in the treatment of both seizures and movement disorders associated with *SLC2A1* [14]. Moreover, triheptanoin showed a sustained improvement on paroxysmal dyskinesia in an open-label trial [96].

PKDs usually respond well to anti-seizure medications (ASMs), though they may resolve spontaneously over time [60]. PKD associated with variants of *PRRT2* is notably sensitive to carbamazepine, but a high response has been reported for other ASMs, such as oxcarbazapine, phenytoin, clonazepam, topiramate, and valproic acid [2]. PNKD attacks usually respond well to benzodiazepines, levetiracetam, and valproic acid [48]. On the contrary, PEDs are poorly responsive to levodopa or ASMs. In these cases, acetazolamide may be beneficial [60].

Symptomatic treatment typically involves pharmacological interventions that modulate the neurotransmitter systems implicated in chorea. Tetrabenazine, a vesicular monoamine transporter 2 (VMAT-2) inhibitor that depletes dopamine from the basal ganglia, represents one of the most used agents in the management of pediatric chorea [60]. Particularly, it provided effectiveness in more than two-thirds of patients with *NKX2-1*-related chorea [97]. Deutetrabenazine and valbenazine, sharing a similar mechanism of action, are also emerging as promising treatments, though evidence supporting their efficacy in pediatric chorea remains limited [60].

A very recent systematic review found that other medications frequently used in the treatment of *NKX2-1*-related chorea are levodopa, methylphenidate, carbamazepine, and topiramate, with benefits described in approximately 53% of patients. The treatment may be initiated early in the course of the disease, especially when severe symptoms are invalidating, and is usually continued for as long as the patient experiences significant benefits with acceptable medication tolerability [98]. On the contrary, low doses of dopamine receptor antagonists were poorly tolerated, inducing an exacerbation of chorea [99].

Tetrabenazine was the mostused and effective drug in *GNAO1*-related chorea, while other commonly used medications only showed a modest effect [71]. However, *GNAO1*-related chorea seems to be progressively refractory to tetrabenazine or antidopaminergic therapy [60]. Deep brain stimulation (DBS) and surgical pallidotomy may be considered promising therapeutic options for patients with *GNAO1*-related chorea in early phases, and particularly in the treatment of exacerbations [4,71].

For *ADCY-5*-related dyskinesias, traditional antidopaminergic therapy often proves ineffective. Instead, improvement has been noted with medications such as clobazam, clonazepam, propranolol, acetazolamide, and methylphenidate [60]. However, heterozygous variants of *ADCY5* associated with paroxysmal episodes have shown a dramatic improvement after treatment with caffeine. Therefore, caffeine should be tried in all patients [100]. In *ADCY5*-related disorders, surgical treatment with DBS is also more beneficial than pharmacotherapy alone [2].

## 6. Conclusions

A wide range of conditions have been related to chorea during infancy and early childhood. These genetic conditions often involve gene pathogenic variants associated with various aspects of neuronal development, synaptic transmission, and neurotransmitter regulation. Deepening our understanding of the molecular mechanisms underlying these disorders allows researchers to gain insights into the pathophysiology of pediatric chorea and identify potential therapeutic targets.

Furthermore, we have highlighted the challenges in managing pediatric chorea, especially in cases where traditional pharmacological therapies may be ineffective or provide only modest relief. Emerging therapies, such as DBS, offer hope for patients with refractory symptoms. However, further research is needed to optimize treatment outcomes.

Overall, a multidisciplinary approach involving geneticists, neurologists, and other specialties is necessary to diagnose and manage pediatric chorea. The ongoing collaboration between clinicians and researchers is essential to advance our understanding of these disorders and improve the lives of affected individuals and their families.

## Figures and Tables

**Table 1:** Genes associated with primary chorea: clinical features, neuroimaging, and treatment options.

Gene	Age at Onset	Inheritance	Chorea Features	Other Hyperkinetic MD	Course of MD	Other Neurologic Features	Cognitive Development	Psychiatric Features	Brain MRI	Treatment Option
*ADCY5*	Infancy (<1 year) to adolescence	AD/de novo	Focal or generalized with facial involvement; Chronic and/or paroxysmal	Episodic exacerbations on awaking, dystonia, and myoclonus	Non-progressive	Axial hypotonia, DD, OMA, alternating hemiplegia, and speech disorders	Normal to mild ID	OCD, depression, anxiety, and phobias	Unspecific	Caffeine, acetazolamide, clonazepam, methylphenidate, and DBS
*chr4p15.3*	Early onset	AR	Generalized; Chronic and/or paroxysmal	HD-like features	Progressive	Extrapyramidal/pyramidal signs, epilepsy, spasticity, and speech disturbances	Cognitive deterioration	NR	Progressive atrophy of the caudates and the frontal cortex	NR
*DRD2*	Infancy (4 months)	De novo	Generalized with facial involvement; Chronic	OMA, occasional myoclonus, and dystonia	Progressive	Axial hypotonia and DD	Mild ID	ADHD, anxiety, and occasional aggressive behavior	Unspecific	Symptomatic: DRD2 antagonists
*GAMT*	Infancy to early childhood	AR	Chronic	Dystonia, ataxia, and hemiballism	NR	DD, hypotonia, epilepsy, spasticity, and severe speech delay	ID and severe learning disability	Autistic-like features and aggressive behavior	Bilateral increased signal intensity in GP	Supplementative
*GCDH*	Infancy (<2 years) to adolescence	AR	Generalized with facial involvement; Chronic	Dystonia and athetosis	Progressive	Macrocephaly, axial hypotonia, DD, epilepsy, acute encephalitic crisis, akinetic-rigid parkinsonism, and speech disorder with dysarthria and apraxia	Normal to ID	NR	Increased signal intensity in the putamen and caudate	Supplementative
*HTT*	Genetic anticipation	AD (CAG repeat expansion)	Sporadic choreic movements; Chronic	Dystonia and impaired saccadic eye movements	Progressive	Bradykinesia, rigidity, parkinsonian features, seizures, and DD or regression	Cognitive deterioration	Depression, suicidal ideation, ODD, and deficit of executive functions	Unspecific	Symptomatic
*JPH3*	Genetic anticipation	AD (CTG-CAG repeat expansion)	Generalized; Chronic	HD-like features	Progressive	Parkinsonism	Cognitive deterioration	Similar to HD	Cerebral atrophy, especially in the caudate and putamen	Symptomatic
*OPA3*	Infancy (1 year) to adulthood	AR	Generalized; Chronic	Ataxia, dystonia, and athetosis	Non-progressive	Hypotonia, DD, optic atrophy, progressive spastic paraplegia, and occasionally epilepsy	Normal and occasionally mild ID	NR	Normal or cerebellar/optic chiasm atrophy	Symptomatic
*PANK2*	Early childhood	AR	Generalized; Chronic	Dystonia and choreoatheosis	Progressive	Rigidity, Spasticity, and speech disorder with dysarthria	Neurodevelopmental regression	NR	“Eye-of-the-tiger” sign and hypointensity of the GP with a central hyperintensity	Symptomatic
*PDE10a*	Infancy (3 months) to early childhood	AD/AR	Generalized, with facial involvement; Chronic and/or paroxysmal	Mild dystonic posturing of upper limbs	Non- or slowly progressive	Axial hypotonia, DD, language delay, dysarthria or stammering, and occasionally epilepsy	Normal to mild ID	NR	Normal or symmetrical bilateral striatal lesions	Symptomatic
*TBP*	Genetic anticipation	AD (CAG/CAA repeat expansion)	Generalized; Chronic and/or paroxysmal	HD-like features, cerebellar ataxia, and dystonia	Progressive	Progressive encephalopathy, progressive akinetic-rigid syndrome, pyramidal signs, and epilepsy	Cognitive deterioration	Psychosis	Atrophy of the cerebellum and caudate nucleus	Symptomatic

Legend: AD: autosomal dominance; ADHD: attention deficit hyperactivity disorder; AR: autosomal recessive; DBS: deep brain stimulation; DD: developmental delay; GP: globus pallidus; HD: Huntington’s disease; ID: intellectual disability; MD: movement disorder; NR: not reported; OCD: obsessive-compulsive disorder; ODD: oppositional-defiant disorder; OMA: oculomotor apraxia.

**Table 2:** Genes causing chorea in the context of epileptic–dyskinetic encephalopathy: clinical features, neuroimaging, and treatment options.

Gene	Age at Onset	Inheritance	Chorea Features	Other Hyperkinetic MD	Course of MD	Other Neurologic Features	Cognitive Development	Psychiatric Features	Brain MRI	Treatment Option
*ATP1A3*	Infancy (<2 years) to adulthood	AD/de novo	Generalized; chronic and/or paroxysmal	Dystonia, myoclonus, ataxia and choreoathetosis, and nystagmus	Progressive	Global DD, hypotonia, microcephaly trigger-induced episodic flaccid tetraplegia, RDP, AHC, and CAPOS syndrome	Moderate ID	Behavioral disorder and executive dysfunction	Unspecific	Symptomatic
*ATP8A2*	Infancy (6 months)	AR	Generalized with facial involvement; chronic	Cerebellar ataxia, athetosis, and dystonia	NR	Global DD, neonatal hypotonia microcephaly, and optic atrophy	Severe ID, with poor/absent speech	ADHD, feeding difficulties, and sleep disorder	Unspecific, cerebral, or cortical atrophy	Symptomatic: tetrabenazine
*FOXG1*	Infancy (<1 year) to early childhood	AD/de novo	Generalized (mainly upper limbs and trunk) with facial involvement; chronic	Dystonia, myoclonus, and hand and/or tongue stereotypies	Non-progressive	Axial or global hypotonia and microcephaly	Severe ID, with absent speech	Sleep disturbances and paroxysmal laughter/crying	CC hypoplasia or aplasia, delayed myelination, simplified gyration, and frontotemporal abnormalities	Symptomatic (pimozide and tetrabenazine)
*FRRS1L*	Infancy (<2 years)	AR	Generalized; chronic	Atethosis, ballismus, and dystonia	Progressive to akinetic-rigid phenotype	Diffuse hypotonia, developmental regression, and rigid- akinetic state	Severe ID	NR	Progressive cortical and cerebellar atrophy	Symptomatic
*GNAO1*	Infancy (<1 year) to early childhood	AD/de novo	Generalized with facial involvement; chronic and/or paroxysmal	Ballismus, dystonia, and episodic exacerbations of chorea/ballism; status dystonicus	Progressive	DD and hypotonia	Severe ID	NR	Unspecific, cerebral atrophy	Symptomatic tetrabenazine, antidopaminergic agents, and DBS
*GRIA2*	Infancy (<1 year)	AD/de novo	Generalized; chronic	Ataxia, dystonia, choreoathetosis, and stereotypies	NR	Gait dyspraxia	Developmental regression, moderate-to-severe ID with poor/absent speech	ADHD, ASD, anxiety, and OCD	Unspecific	Symptomatic: ASMs
*GRIA3*	Early childhood (<3 years)	X-linked	Generalized; chronic	Multifocal myoclonus and dystonia	Non-progressive	Exaggerated startle reflex, language delay, and dysarthria	Mild ID	Occasional ODD, ADHD, and anxiety	Unspecific	Symptomatic: ASMs and tetrabenazine
*GRIN1*	Infancy (<1 year)	AD/AR	Generalized; chronic	Dystonia, stereotypies, and oculogyric crises	Non-progressive	Cortical visual impairment spastic tetraplegia, hypotonia	Mild-to-severe ID with absent speech	Behavioral disorder, sleep disturbances, and ASD	Unspecific, cerebral atrophy and polymicrogyria	Symptomatic: ASMs
*GRIN2D*	Infancy (<1 year)	AD/de novo	Generalized; chronic	Dyskinetic and choreiform movements	NR	DD/ID, hypotonia, tetraplegia, and cortical visual impairment	Mild-to-severe ID	Sleep disturbances and ASD	Unspecific, cerebral atrophy	Symptomatic: ASMs
*SYT1*	Early childhood (3 years) to adolescence	AD/de novo	Generalized, especially of the lower limbs; chronic and/or paroxysmal	Mixed movement: dystonia, ballism, athetosis, and nocturnal episodic exacerbations	NR	Hypotonia, delayed visual maturation, and severe DD	Severe ID	Episodic agitation and sleep disorder	Unspecific	Symptomatic: dopamine agonist (pramipexole)
*UNC13A*	Congenital	De novo	Generalized, especially of the upper limbs; chronic and/or paroxysmal	Dyskinesias with intention tremor and rare febrile seizures	Non-progressive	Developmental and speech delay and hypotonia	Mild ID	ASD and ADHD	Unspecific	Symptomatic

Legend: AD: autosomal dominance; ADHD: attention deficit hyperactivity disorder; AHC: alternating hemiplegia of childhood; AR: autosomal recessive; ASD: autism spectrum disorder; ASMs: anti-seizure medications; CAPOS: cerebellar ataxia, areflexia, pes cavus, optic atrophy, and sensorineural hearing loss; CC: corpus callosum; DBS: deep brain stimulation; DD: developmental delay; ID: intellectual disability; MD: movement disorder; NR: not reported; OCD: obsessive-compulsive disorder; ODD: oppositional-defiant disorder; RDP: rapid-onset dystonia parkinsonism.

**Table 3:** Genes causing chorea associated with other multiorgan manifestations: clinical features, neuroimaging, and treatment options.

Gene	Age at Onset	Inheritance	Chorea Features	Other Hyperkinetic MD	Course of MD	Other Systemic Features	Cognitive Development	Neuropsychiatric Features	Brain MRI	Treatment Option
*ATM*	Infancy (1 year) to adolescence	AR	Generalized; chronic	Prominent ataxia, mixed MD: myoclonus, choreoathetosis, and dystonia	Progressive	Ocular telangiectasias, immunodeficiency, and a predisposition to malignancy	Cognitive deterioration	Peripheral neuropathy, oculomotor apraxia, and dysarthria	Unspecific	Symptomatic: Antiglutamatergic agent: Amantadine
*FXN*	Genetic anticipation	AR (GAA repeat expansion and/or mutations)	Generalized; chronic	Dystonia, ataxia and macrosaccadic oscillations	Progressive	Pes cavus, diabetes mellitus, cardiomyopathy, and scoliosis	NR	Areflexia, peripheral sensory loss, and extensor plantar reflexes	Unspecific	NR
*NKX-2.1*	Infancy (<1 year) to early childhood	AD/de novo	Generalized; chronic, worsen with stress or excitement	Myoclonus, dystonia, ataxia, and motor/vocal tics	Non-progressive or improve with age	Hypothyroidism, thyroid carcinoma, pulmonary infections, neonatal RDS, obstructive airway disorders, interstitial lung disease, and lung cancer	Almost normal	Hypotonia, DD, ASD, ADHD, anxiety, and executive dysfunction	Unspecific	Symptomatic: Levodopa, tetrabenazine, methylphenidate
*SLC16A2*	Congenital or within 6 months	X-linked	Generalized; chronic and/or paroxysmal	Dystonia, choreoathetosis, paroxysmal dyskinesia	Progressive to spastic paraplegia	Hypothyroidism and lung cancer	ID	Hypotonia and global DD	Unspecific	Symptomatic

Legend: AD: autosomal dominance; ADHD: attention deficit hyperactivity disorder; AR: autosomal recessive; ASD: autism spectrum disorder; DD: developmental delay; ID: intellectual disability; MD: movement disorder; NR: not reported; RDS: respiratory distress syndrome.
